# fMRI BOLD Correlates of EEG Independent Components: Spatial Correspondence With the Default Mode Network

**DOI:** 10.3389/fnhum.2018.00478

**Published:** 2018-11-27

**Authors:** Marcel Prestel, Tim Paul Steinfath, Michael Tremmel, Rudolf Stark, Ulrich Ott

**Affiliations:** Bender Institute of Neuroimaging, Justus Liebig University Giessen, Giessen, Germany

**Keywords:** fMRI, EEG, independent component analysis, EEG independent component clustering, default mode network

## Abstract

**Goal**: We aimed to identify electroencephalographic (EEG) signal fluctuations within independent components (ICs) that correlate to spontaneous blood oxygenation level dependent (BOLD) activity in regions of the default mode network (DMN) during eyes-closed resting state.

**Methods**: We analyzed simultaneously acquired EEG and functional magnetic resonance imaging (fMRI) eyes-closed resting state data in a convenience sample of 30 participants. IC analysis (ICA) was used to decompose the EEG time-series and common ICs were identified using data-driven IC clustering across subjects. The IC time courses were filtered into seven frequency bands, convolved with a hemeodynamic response function (HRF) and used to model spontaneous fMRI signal fluctuations across the brain. In parallel, group ICA analysis was used to decompose the fMRI signal into ICs from which the DMN was identified. Frequency and IC cluster associated hemeodynamic correlation maps obtained from the regression analysis were spatially correlated with the DMN. To investigate the reliability of our findings, the analyses were repeated with data collected from the same subjects 1 year later.

**Results**: Our results indicate a relationship between power fluctuations in the delta, theta, beta and gamma frequency range and the DMN in different EEG ICs in our sample as shown by small to moderate spatial correlations at the first measurement (0.234 < *|r|* < 0.346, *p* < 0.0001). Furthermore, activity within an EEG component commonly identified as eye movements correlates with BOLD activity within regions of the DMN. In addition, we demonstrate that correlations between EEG ICs and the BOLD signal during rest are in part stable across time.

**Discussion**: We show that ICA source separated EEG signals can be used to investigate electrophysiological correlates of the DMN. The relationship between the eye movement component and the DMN points to a behavioral association between DMN activity and the level of eye movement or the presence of neuronal activity in this component. Previous findings of an association between frontal midline theta activity and the DMN were replicated.

## Introduction

Synchronous low frequency fluctuations in the blood oxygenation level dependent signal (BOLD) as measured by functional magnetic resonance imaging (fMRI) during rest have gained considerable interest in recent years (Raichle, 2011). These temporally correlated and spatially organized large-scale resting state networks (RSNs) can be detected in the absence of a specific task, are therefore easily obtained from different populations (e.g., children or patients), and are consistently identified across subjects (Beckmann et al., 2005; Fox and Raichle, 2007). In addition, numerous neurological and psychiatric disorders were shown to be associated with altered RSN activity and connectivity (Menon, 2011; Mulders et al., 2015).

Until now, 10–20 RSNs have been consistently identified (Kalcher et al., 2012; Allen et al., 2018), while especially the task negative default mode network (DMN) received substantial attention. Regions of the DMN have a high energy demand (Raichle, 2011), receive more blood flow (Zou et al., 2009), and are characterized by their high connectivity (Hagmann et al., 2008) indicating the importance as network of information integration (van den Heuvel et al., 2012). Functionally, the DMN has been associated to operations such as self-referential, emotional and perceptual processing (Mason et al., 2007; Coutinho et al., 2016). Methodologically, RSNs are commonly identified using data-driven independent component analyses (ICA), which is frequently applied as an unsupervised learning method that separates mixed signals into maximally statistically independent components (ICs; Damoiseaux et al., 2006).

While the DMN has been of major interest in cognitive neuroscience in the last decade, the underlying neuronal activity remains incompletely understood due to the indirect nature of the hemeodynamic signal measured by fMRI. This is in contrast to the signal measured by electroencephalographic (EEG), which is produced by superposition of synchronous neuronal electrical activity primarily originating from cortical pyramidal cells (David and Vince, 2014). At the electrodes on the scalp, activity from concurrently active brain sources and non-brain related signals are recorded as mixed signals through volume conduction (Delorme et al., 2012). As potential solutions for unmixing the signals, ICA is popular in EEG research since it allows the reconstruction of the different source activity time courses by maximizing their statistical independance. However, it remains difficult to identify common sources across subjects due to the lack of correspondence in the time courses, inter-individual variability of scalp topographies, and unstable ICA results (Huster et al., 2015). Nonetheless, ICA can lead to physiologically plausible components based on the assumption that the underlying sources can be modeled as dipoles (Palmer et al., 2011; Delorme et al., 2012) and similar components can be identified across subjects by application of clustering algorithms (Delorme and Makeig, 2004). In addition, compared to other approaches to reconstruct source activity that assume a physical head model, ICA has no assumptions about the underlying physiological structures and is unsusceptible to individual differences that violate these assumptions. Simultaneous recordings of fMRI and EEG have the potential to improve our understanding of the resting state brain activity and can be used to investigate whether the components of the different imaging modalities converge.

Several studies have investigated the relationship between the resting state fMRI and EEG signal by correlating the time course of EEG frequency band power with the BOLD signal. Special emphasis was placed on the BOLD correlates of the alpha rhythm recorded from posterior electrodes, where one of the most prominent findings is a negative correlation between alpha activity and posterior regions of the visual cortex (Laufs et al., 2003a,b; Moosmann et al., 2003; Feige et al., 2005; Gonçalves et al., 2006). In addition, the correlation between other EEG rhythms and their interactions with the BOLD signal revealed different associations with mixed results (Laufs et al., 2003b; Scheeringa et al., 2008; Tyvaert et al., 2008; de Munck et al., 2009; Marawar et al., 2017). In particular, it was shown that the relationship between EEG band power fluctuations and the fMRI BOLD signal are subjected to large inter- and intra-individual variability during resting state (Gonçalves et al., 2006; Meyer et al., 2013). This highlights the importance of replication studies that investigate the reproducibility of EEG-fMRI associations over time. With regard to the DMN, frequency band specific correlations to the BOLD signal were shown in several studies. During eyes closed rest, regions of the DMN are positively correlated to beta band fluctuations (Laufs et al., 2003b). In case of alpha activity, the association to the DMN appears to be state dependent, since a relationship to the DMN was shown only during eyes open rest, but not eyes closed rest (Scheeringa et al., 2012; Mo et al., 2013). In addition, theta activity from a frontal EEG component is negatively correlated to the DMN during eyes open rest (Scheeringa et al., 2008). While these studies indicated an association of single frequencies to BOLD activity in regions associated with the DMN, it was also shown that RSNs are characterized by specific mixtures of EEG rhythms, which might explain the findings in such diverse frequency bands (Mantini et al., 2007).

Due to the fact that EEG measured at the scalp is a mixture of several volumes conducted cortical sources, the origin of the EEG signal, which correlates to the BOLD signal fluctuations, remains incompletely understood. When studying the relationship between EEG rhythms and the BOLD signal on the group-level it appears that large inter-individual variability in this association leads to inconsistent results (Gonçalves et al., 2006; Tyvaert et al., 2008). Since most studies have investigated EEG signal fluctuations on the scalp level, focusing on EEG source separated signals may lead to more robust EEG-BOLD relationships across subjects. By analyzing simultaneously recorded resting state EEG/fMRI we aimed to examine whether EEG rhythms of source separated signals correlate with BOLD fluctuations in regions of the DMN. In addition, we investigated the reproducibility of the results over time by repeating the analyses with data from the same subjects recorded 1 year later. We hypothesized, that stable and reproducible activity within regions of the DMN can be derived from EEG data which closely resembles the DMN obtained by fMRI-ICA in the spatial domain. Especially in the light of pathophysiological alterations in RSNs commonly observed in various disorders, the resulting EEG signatures could be used in therapeutic interventions aiming to specifically influence localized brain activity in real-time (e.g., neurofeedback; Rogala et al., 2016).

## Materials and Methods

### Participants

The convenience sample used in this study consisted of 20 experienced meditators (10 females; mean age ± SD: 46.96 ± 12.46 years) participating in a meditation training program (Timeless Wisdom Training, TWT) and 10 controls (five females; mean age ± SD: 41.44 ± 14.44 years) without any meditation experience. TWT participants and controls were scanned before the training started and after the first year of the program. These two time-points of measurement will be referred to as time-point 1 and time-point 2. Both groups were comparable with respect to their educational background with 85% of all TWT participants and 90% of all controls having a degree from a university of applied sciences or higher. Participants were predominantly right-handed, with one subject from the TWT group being left-handed. The study was carried out according to the ethical guidelines of the review board of the German Psychological Society (DGPs; reference number: DVUO29092008DGPS) with written informed consent from all subjects in accordance with the Declaration of Helsinki. The protocol was approved by the review board of the German Psychological Society. Participants received reimbursement of travel expenses and a financial compensation for participation.

### Scanning Procedure

The scanning session was identical at both time-points and divided into two parts. First, an anatomical scan was taken lasting for around 6 min. Subsequently, the EEG cap and additional peripheral sensors were attached outside of the scanner. A 40-min functional scan was performed consisting of two 20-min blocks, a resting state condition, where the participants were asked to let their mind wander freely with the eyes closed, followed by a mindfulness meditation condition, where the subjects focused their attention on the sensations arising around the nostrils during breathing. Furthermore, the subjects were instructed to not change their breathing pattern and return their focus on the breath once they realized an episode of mind wandering. The second block is not included in the current analysis.

### EEG Data Acquisition

EEG was recorded simultaneously inside the MRI scanner from the subject’s scalp using 30 sintered Ag/AgCl electrodes attached to an EEG cap (BrainCap-MR 32 Channels, Easycap, Hersching, Germany) placed according to the 10-20 system and MRI-compatible BrainAmp MR amplifiers (Brain Products GmbH, Munich, Germany). The sampling rate was 5 kHz and the signal was referenced to an electrode positioned between Fz and Cz. Impedance was kept below 5 kΩ. Two remaining channels were used for electrocardiogram (ECG, attached to the subject’s back) and electrooculogram (EOG, attached besides the left eye). The helium-pump of the MR scanner was turned off during acquisition to reduce the generation of additional artifacts. Synchronization of the EEG and fMRI gradient system clocks was achieved via the Brain Products SyncBox (Brain Products, Munich, Germany) to ensure accurate gradient artifact sampling and improvement of the subsequent gradient artifact subtraction. The beginning of each MR volume was automatically marked in the EEG data.

### (f)MRI Data Acquisition

From each participant, functional and structural MRI images were acquired using a Siemens Symphony (Erlangen, Germany) 1.5-Tesla MR scanner and a standard 1-channel head coil for radiofrequency transmission and reception. A high-resolution three-dimensional magnetization prepared rapid acquisition gradient echo (3D MPRAGE) T1-weighted anatomical scan was acquired from each subject with the following parameters: 160 slices, time to repetition (TR) = 1990 ms, echo time (TE) = 4.18 ms, flip angle (FA) = 15°, matrix size = 256 × 256, Field of View (FOV) = 250 × 250 mm^2^, ~1 × 1 × 1 mm^3^ isotropic voxels. A gradient echo field map sequence was obtained with an echo time difference of 4.76 ms. Eight-hundred and ten axial whole brain functional volumes were acquired in a descending order using a T2* weighted gradient-echo-planar-imaging (EPI) sequence. The following pulse-sequence parameters were used: TR = 3,000 ms, TE = 50 ms, FA = 90°. EPI volumes were acquired at 30 slices with 4 mm thickness and a 1 mm slice gap. Matrix size was 64 × 64, FOV = 192 × 192 mm^2^ and voxel size = 3 × 3 × 4 mm^3^. Slices were aligned parallel to the anterior-posterior commissure plane.

### Physiological Recording

Respiration was measured using a respiratory belt positioned at the level of the costal arch measuring expansions during breathing. Cardiac activity was measured with electrocardiography (ECG) and pulse frequency was assessed using a finger clip.

### EEG Preprocessing

**Figure 1:**
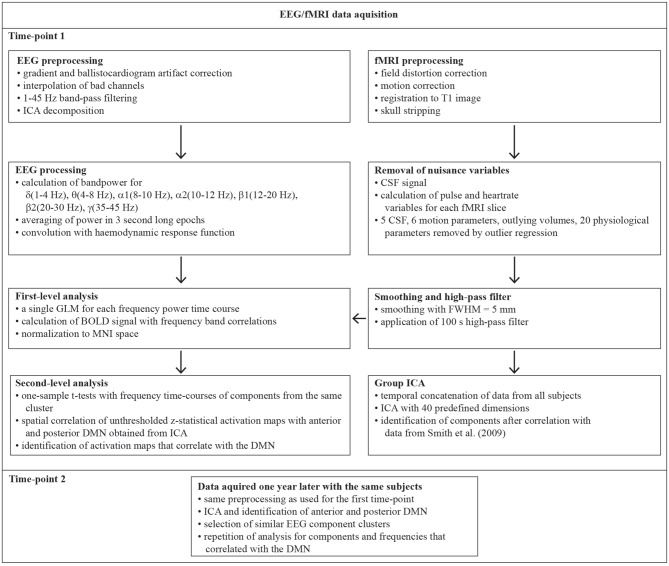
Schematic overview of the functional magnetic resonance imaging-electroencephalographic (fMRI-EEG) processing pipeline. The two time-points were 1 year apart. ICA, Independent Component Analysis; CSF Cerebrospinal fluid; FWHM, Full Width Half Maximum; GLM, General Linear Model; MNI, Montreal Neurological Institute; BOLD, Blood Oxygenation Dependent Signal; DMN, default mode network.

A schematic overview of the analyses performed is shown in Figure 1. Brain Vision Analyzer 2 (BrainProducts, Germany) was used to account for timing jitter of the MR scanner volume marker timing. All markers were moved to a distinctive element of the artifact by −15 ms, the data sampling rate was increased by factor 10 and the Brain Vision Analyzer function Slice Volume Align was applied for marker realignment. Subsequent gradient artifact removal was performed by subtracting an artifact template from the data, using a baseline-corrected sliding average of 21 consecutive volumes (Allen et al., 2000) followed by down-sampling to 500 Hz. In the next step, ballistocardiogram (BCG) related artifacts were removed by subtraction of averaged EEG signal in synchrony to the heartbeat events (R peaks) as measured with the ECG (Allen et al., 1998). The data was imported to EEGLAB (Delorme and Makeig, 2004,^1^), running under the Matlab R2015a environment. Continuous EEG data was downsampled to 250 Hz and a 1–45 Hz band-pass FIR filter was applied to remove low-frequency drifts and high-frequency noise. Artifact dominated channels and data segments were identified and removed by application of the artifact subspace reconstruction method as implemented in the clean_rawdata plugin of EEGLAB. Removed channels were interpolated to reduce potential biases in the re-referencing step. The EEG signal was then re-referenced to the common average and Adaptive Mixture Independent Component Analysis (AMICA; Palmer et al., 2011) was used to temporally decompose a 12 min data sequence ranging from 7 min to 9 min corresponding to the resting state fMRI data. We chose the AMICA algorithm since it performs significantly better than other ICA and blind source separation approaches in the generation of dipolar EEG components while also producing the greatest reduction in mutual information (Delorme et al., 2012). We used the Dipfit2 algorithm as implemented in EEGLAB for the location of equivalent dipoles associated with each IC in the MNI standard space. Since ICs are thought to reflect locally restricted synchronous cortical activity that can be explained by an equivalent dipole, the amount of spatial variance of an IC explained by its associated equivalent dipole can serve as quality criterion for the selection of EEG components. Hence, if dipoles accounted for less than 85% of the ICs spatial variance or were located outside of the brain, the associated ICs were excluded from further analysis since they were likely to be associated with non-brain sources. These restrictions led to the inclusion of 550 components for time-point 1 (61.1%, 18.3 per subject) and 576 (64%, 19.2 per subject) for time-point 2.

### Independent Component Clustering

In order to investigate comparable ICs from the temporal decomposition of single-subject data across-subjects, the k-means clustering algorithm as implemented in EEGLAB was applied to the individual ICs using the following parameters: location of the equivalent dipole (dimensions: 3, relative weighting 10), mean log power spectrum (1–45 Hz, dimensions: 10, weighting: 2) and scalp maps (dimensions: 7, weighting: 3). We aimed to emphasize the spatial correspondence of the components, hence the highest weights were given to dipole locations and scalp maps. Due to inter-individual variability in gyrification pattern, the dipole direction and associated scalp topography can vary drastically. Hence, the largest weights were given to dipole location, without taking their orientation into account. The number of clusters was predefined to be 19, so that on average each subject had the chance to contribute one IC to each cluster.

### fMRI Preprocessing

SPM12^2^ and FSL 5.0.9 (FMRIB’s Software Library^3^) were used for preprocessing of functional and structural MRI data. Due to excessive EEG artifacts, nine volumes from the beginning and one volume at the end of the fMRI time-series were removed from the data, resulting in a total of 800 functional volumes.

B0-field-distortion correction was performed using field map based realign and unwarping (SPM12) with the first volume as reference.

Outlier detection was performed on a volume by volume basis by calculating the mean squared differences to the previous and the next volume. The smaller difference was used as deviation score for each volume. The scores were thresholded using the method of Hubert and Van der Veeken (2008) which calculates a robust measure of skewness (Medcouple, MC, USA) and uses it for correcting the interquartile range (IQR). For thresholding deviation scores, the IQR was multiplied by 1.5 and added to the 75th percentile (P75). Subsequently, outlying volumes were corrected for in a nuisance regression step (see below) using FSL FEAT by incorporation of an additional regressor for each outlying volume.

Unwarped functional images were co-registered to subject specific high-resolution anatomical brain images that were segmented into gray matter (GM), white matter (WM) and cerebrospinal fluid (CSF) using segment from SPM12 and skull-stripped using the ROBEX algorithm Iglesias et al., 2011).

CSF clusters in functional volumes were determined by resampling the T1 based CSF mask to the functional series. Time-series were extracted from five largest CSF clusters using the first eigenvariate.

Physiological noise regressors were created for each functional series based on peripheral cardiac and respiration recordings using the PhysIO Toolbox Kasper et al., 2017). Specifically, retrospective image-based correction (RETROICOR; Glover et al., 2000) with application of Fourier expansion for the cardiac phase (3rd order), respiration (4th order) and cardio-respiratory interactions (1st order), respiration volume per unit time (RVT; Birn et al., 2013) and heart rate variability (HRV; Chang et al., 2009) were calculated resulting in 20 regressors.

Subsequently, identified outlying volumes and a total of 31 nuisance regressors corresponding to cardiac, respiration, CSF and motion were removed from the fMRI time courses using FSL’s (5.0.9) FEAT (Jenkinson et al., 2002). The mean functional image was added to the remaining residuals.

A total of 12 min (7–19 min) from the resting condition were selected to allow for optimal ICA data decomposition (Birn et al., 2013) while reducing the computational demand of the analyses. Smoothing with a 5 mm full-width half maximum (FWHM) Gaussian kernel was applied and the data were high-pass filtered at 100 s.

### Group Independent Component Analysis (group ICA)

Registration of individual functional data to high resolution MNI125 standard space images was carried out using FSL FLIRT (Jenkinson and Smith, 2001; Jenkinson et al., 2002). Group ICA was performed following a temporal concatenation approach of the single subject data, using Probabilistic Independent Component Analysis (Beckmann and Smith, 2004) as implemented in FSL’s Multivariate Exploratory Linear Decomposition into ICs (MELODIC) version 3.14. The data of all 30 participants over both time-points were concatenated and a spatial decomposition with 15 predefined dimensions was used. The number of predefined dimensions was determined by the maximum number at which the DMN was not split into two separate ICs. The ICs were labeled after spatial correlation with the exemplar dataset provided by Smith et al. (2009)^4^ using the FSL-tool fslcc for calculation. In this way, the DMN used in later analyses was obtained.

### Test for Differences Within the Sample Regarding Levels of Meditation Experience

In order to test whether the DMN differed between subjects with different amounts of meditation experience, FSL dual-regression analysis was performed. Subjects were assigned to three groups: controls (10 subjects; no meditation experience), intermediate meditators (10 subjects; less than 11 years of meditation experience; M = 2.02 years; SD = 3.15 years) and expert meditators (10 subjects; more than 11 years of meditation experience; M = 19.5 years; SD = 6.45 years). The DMN obtained from the group ICA analysis was used to generate subject-specific versions of the spatial maps, and associated timeseries, using dual regression (Beckmann et al., 2009; Nickerson et al., 2017). First, for each subject, the DMN spatial map is regressed (as spatial regressors) into the subject’s 4D space-time dataset. This results in a subject-specific timeseries associated with the DMN. Next, this timeseries is regressed (as temporal regressor) into the same 4D dataset, resulting in a spatial map reflecting the subject-specific DMN. We then tested for between group differences via F-tests using FSL’s *randomize* permutation-testing tool with threshold-free cluster enhancement and 10,000 permutations.

### EEG Regressor Computation and General Linear Model Analysis

The time course of each IC was decomposed by Fast Fourier Transform (FFT) into seven non-overlapping frequencies bands (delta: 1–4 Hz, theta: 4–8 Hz, alpha1: 8–10 Hz, alpha2: 10–12 Hz, beta1: 12–20 Hz, beta2: 20–30 Hz, gamma: 35–45 Hz). Within each frequency the power was averaged and the time course subdivided into 3 s long segments corresponding to the recorded fMRI volumes. The resulting time series were convoluted with a double gamma hemeodynamic response function (HRF) as implemented in FSL FEAT. Hence, we obtained one regressor per frequency band for each EEG IC. Since the different frequency band power fluctuations can be significantly correlated, we created one GLM for each frequency to capture the whole extent of correlated BOLD activity at the cost of lower specificity, as compared to models, where the covariation between frequency bands is accounted for (de Munck et al., 2009; Tyvaert et al., 2008). The obtained statistical parametric maps were registered to 4 mm MNI125 standard space using FSL FLIRT. On the group-level, one-sample *t*-tests were computed using FSL FMRIB Local Analysis of Mixed Effects (FLAME) for each frequency band and EEG cluster leading to 133 group-level analyses (19 (EEG clusters)* 7 (frequency bands)) that were performed for the first time-point. After identification of z-score correlation maps of interest, respective analyses were repeated at the second time-point for 10 IC cluster and frequency band combinations.

### Comparison of GLM- and Group ICA Analyses

The spatial correlation between the DMN component and the regression results was calculated using fslcc, in which a mask was used that left only gray matter voxels. We included negative as well as positive *z*-values from the regression results and the DMN map in the spatial correlation analysis. To select only correlation maps that show some spatial overlap with our example DMN, a cutoff value of more than 5% variance explained corresponding to a correlation coefficient of 0.224 was used. The remaining maps are not considered in this analysis. Significance of the correlations was assessed at a Bonferroni-adjusted *p* = 0.000376 (0.05/133 comparisons). The analysis was repeated with data acquired 1 year later from the same subjects for component clusters that met the defined correlation threshold. Component Clusters 7 and 15 could not be identified at the second time-point.

## Results

### Group fMRI ICA Analysis and Test for Group Differences

**Figure 2:**
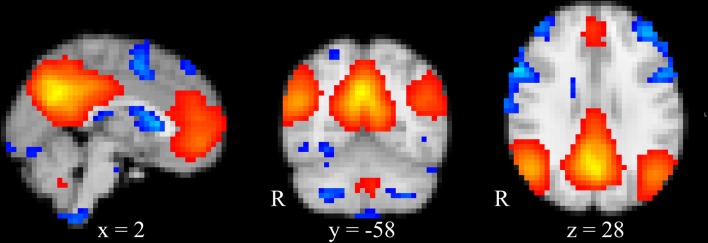
Group ICA estimated DMN. DMN obtained from temporally concatenated group ICA. Images are superimposed onto the MNI standard brain. Blue indicates z-scores below the 10th percentile and red indicates z-scores above the 90th percentile.

The estimated independent component from the group ICA decomposition with the highest correlation (*r* = 0.81) to the DMN found by Smith et al. (2009) is depicted in Figure 2.

The groups based on different durations of meditation experience did not differ significantly in their DMN ICs. The F-tests comparing the group means for time-point 1 showed that all familywise error-corrected *p* > 0.36 and for time-point 2 all *p* > 0.9.

### EEG Clustering

We identified common EEG components across the group using k-means clustering based on their scalp topography, dipole location, and frequency spectrum. At both time-points, 19 IC’s were retained based on the average number of available ICs across subjects. The average percentage of subjects contributing to each IC Cluster was 68.4% at TP1 and 66.7% at TP2 with around 1.5 components contributed to each cluster from each subject (see Table 1). No significant differences between time-points with regard to the amount of subjects contributing to each cluster or the amount of components being assigned to each cluster were present (Student’s *t*-test, *p* > 0.05).

**Table 1:** Independent component (IC) clustering of electroencephalographic (EEG) data.

	Time-point 1		Time-point 2	
Cluster	#Subjects	#Components	#Subjects	#Components
1	21	34	20	39
2	23	36	19	24
3	17	24	18	19
4	28	42	25	38
5	21	31	20	26
6	16	17	25	36
7	15	17	25	43
8	25	38	19	28
9	15	18	15	29
10	23	37	14	25
11	24	40	22	36
12	18	19	27	41
13	20	29	21	25
14	19	30	18	23
15	19	20	18	22
16	20	27	18	27
17	27	39	20	34
18	20	31	18	27
19	19	21	18	34
Mean	20.5 (68.4%)	28.9 (1.4/S)	20 (66.7%)	30.3 (1.51/S)
SD	3.7	8.5	3.5	7.1

*Summary of the number of ICs and the number of subjects per time-point contributing to each IC cluster*.

### EEG Frequency Band Power GLM Analysis

After identification of common EEG IC’s across subjects, we addressed the question whether EEG IC time courses are correlated to the simultaneously recorded BOLD signal in a frequency specific manner. Hence, each frequency band power time-course was used in a single session level GLM and group analyses were performed using all components assigned to each cluster. We identified EEG component and frequency band combinations which correspond to the DMN by spatially correlating the z-score map of the DMN with the results from the regression analyses.

**Figure 3:**
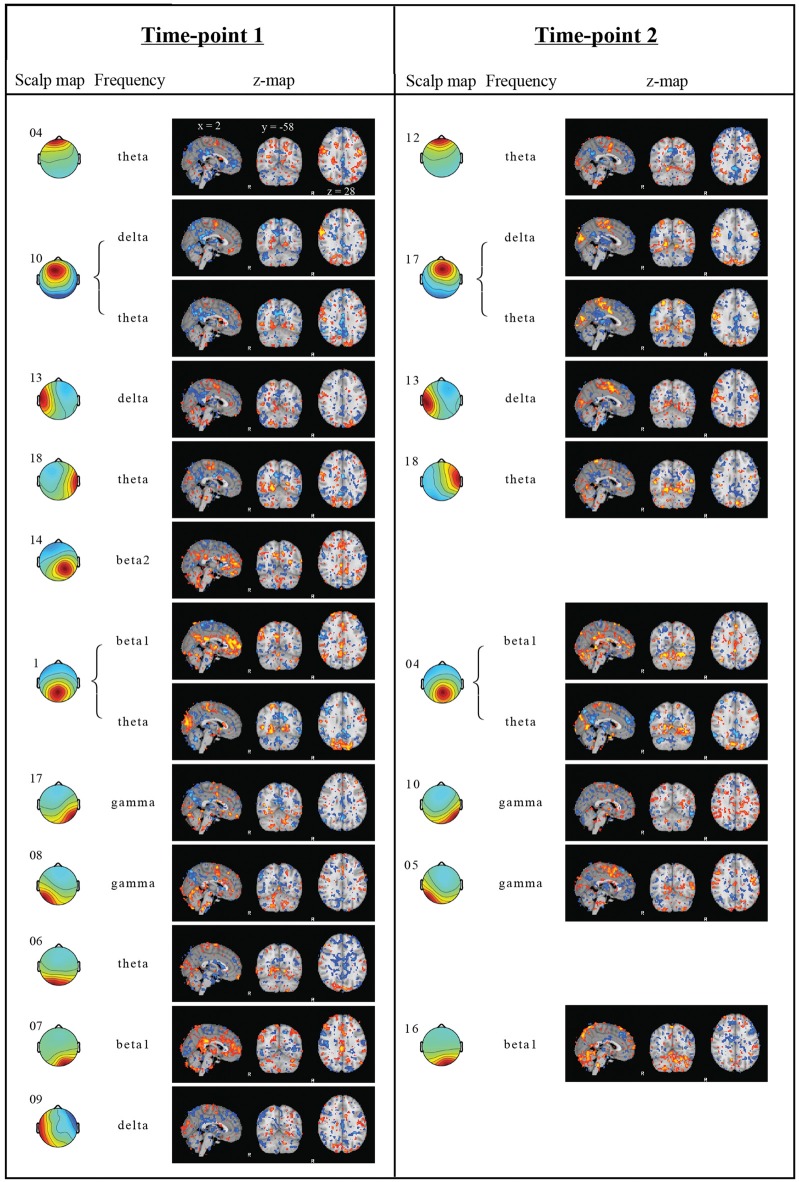
Regression results. Independent components (IC) scalp map with cluster number, frequency band and correlation maps from both time-points. EEG scalp maps indicate, which EEG electrodes contribute to the IC. Only regression analysis results, which explain more than 5% of the example DMN spatial variance in a voxelwise correlation, are shown. Z-score maps obtained from the GLM regressions were thresholded below the 10th percentile (blue) and above the 90th (red). The correlation coefficients between corresponding correlation maps from time-point 1 and 2 ranged from −0.03 to 0.52 (mean 0.28).

Figure 3 displays the fMRI correlation maps, which correspond to specific frequencies obtained from EEG ICs at the two time-points. For illustration purposes, in Figures 2, 3, correlation maps were individually thresholded to show correlations below the 10th percentile and above the 90th percentile. Table 2 lists the correlations between correlation maps of cluster and frequency combinations from time-point 1 with the DMN obtained from group ICA. A total of 13 correlation maps correlated above our defined threshold and absolute correlations ranged from *r* = 0.234 (Cluster 9, *t* = 38.0, *p* < 0.0001) to *r* = 0.346 (Cluster 17, *t* = 58.2, *p* < 0.0001). Interestingly, most frequencies were negatively associated with activity in regions of the DMN, while only for the beta band we found a positive relationship and no association in case of both alpha frequencies. Only the Clusters 1 and 10 had more than one correlation map that correlated above the threshold. In case of Cluster 1 the correlation maps of the theta and beta1 frequency were correlated with the DMN in different directions: while the map associated with the beta1 frequency showed a positive correlation with the DMN (*r* = 0.278, *t* = 45.7, *p* < 0.0001), the map corresponding to the theta range had a negative one (*r* = −0.278, *t* = 45.7, *p* < 0.0001). Surprisingly, a frontal EEG cluster commonly associated with eye movements was correlated to BOLD activity in regions of the DMN (Cluster 4, *r* = −0.249, *t* = 40.6, *p* < 0.0001).

**Table 2:** Clusters at time-point 1.

Cluster	Frequency	Correlation	*t*-value	*p*-value
17	gamma	−0.345	58.2	<0.0001
10	theta	−0.332	55.7	<0.0001
13	delta	−0.291	48	<0.0001
1	beta1	0.278	45.7	<0.0001
1	theta	−0.278	45.7	<0.0001
14	beta2	0.269	44.1	<0.0001
18	theta	−0.26	42.5	<0.0001
8	gamma	−0.253	41.3	<0.0001
4	theta	−0.249	40.6	<0.0001
6	theta	−0.249	40.6	<0.0001
7	beta1	0.243	39.6	<0.0001
10	delta	−0.238	38.8	<0.0001

*Spatial correlation between group-level correlation maps at time-point 1 and the group ICA estimated default mode network (DMN)*.

### Replication at Time-Point 2

In order to investigate whether the obtained results are reliably detected, the analyses were repeated with data obtained from the same subjects 1 year later. Only cluster and frequency combinations which resulted in correlation maps correlated with the DMN at time-point 1 were considered. Component clusters at time-point 2 were selected based on visual similarity with clusters from time-point 1. No corresponding maps for the Clusters 6, 9 and 14 of time-point 1 could be identified at time-point 2 (Figure 3). Table 3 lists correlation values of the DMN to correlation maps resulting from the GLM analysis.

**Table 3:** Clusters at time-point 2.

Cluster	Frequency	Correlation	*t*-value	*p*-value
10	gamma	0.0675	10.7	<0.0001
17	theta	−0.223	36.1	<0.0001
13	delta	−0.17	27.2	<0.0001
4	beta1	0.178	28.6	<0.0001
4	theta	−0.275	45.2	<0.0001
18	theta	−0.161	25.7	<0.0001
5	gamma	−0.158	25.3	<0.0001
12	theta	−0.283	46.7	<0.0001
16	beta1	0.0138	2.18	0.0292
17	delta	−0.226	36.7	<0.0001

*Spatial correlation between group-level correlation maps at time-point 2 and the group ICA estimated DMN*.

In general, correlations were lower at time point 2, but only the two following results from time-point 1 could clearly not be replicated. The initial correlation of *r* = 0.243 (*t* = 39.6, *p* < 0.0001) of the beta1 frequency from Cluster 7 with the DMN at time-point 1 dropped in the corresponding Cluster 16 at time-point 2 to *r* = 0.014 (*t* = 2.18, *p* = 0.0292). In addition, Cluster 18 was initially correlated to the DMN with *r* = −0.346 (*t* = 58.2, *p* < 0.0001), but the corresponding Cluster at the second time-point did not replicate this finding (Cluster 10, *r* = 0.068, *t* = 10.7, *p* < 0.0001).

The absolute correlations of the remaining correlation maps at time-point 2 are in the range from *r* = 0.158 (Cluster 6, *t* = 25.3, *p* < 0.0001) to *r* = 0.283 (Cluster 13, *t* = 46.7, *p* < 0.0001). Parietal theta seemed to replicate best with *r* = −0.278 (Cluster 1, *t* = 45.7, *p* < 0.0001) at time-point 1 and *r* = −0.275 (Cluster 5, *t* = 45.2, *p* < 0.0001) at time-point 2. In conclusion, 8 of 13 Clusters were replicated at the second time-point.

## Discussion

The aim of the present study was to investigate whether frequency specific signal fluctuations of EEG ICs are correlated with BOLD signal changes within regions of the DMN during the eyes-closed resting state. We have found an association between the power of several frequency bands of different EEG ICs with BOLD signal fluctuations in regions that are spatially correlated with the DMN. In particular, this relationship was observed for the delta, theta, beta and gamma frequency band in different EEG ICs. Furthermore, theta activity within an EEG component commonly interpreted as eye movements correlated negatively with BOLD activity in regions of the DMN. These correlations appear to be in part reproducible over time, since 8 out of 13 EEG clusters showed an association with the DMN in the same subjects 1 year later.

### EEG IC Power Fluctuations Correlate to the BOLD Signal in Regions of the DMN

The findings of this study suggest a complex relationship between the representation of the DMN found in the fMRI-BOLD signal and EEG component activity. Not only was activity from EEG IC Clusters found to correlate with the DMN, this correlation was also present within different frequency bands in positive as well as negative directions. These findings further strengthen the results previously reported in literature, where associations between the DMN and the theta (Scheeringa et al., 2008; Tyvaert et al., 2008; Marawar et al., 2017), delta (Hlinka et al., 2010) and beta frequency bands (Laufs et al., 2003b; Moosmann et al., 2003; Hlinka et al., 2010; Neuner et al., 2014) were observed. In addition, our results indicate a negative relationship between high-frequency brain activity in the gamma frequency range (35–45 Hz) and spontaneous BOLD signal fluctuations in the DMN. While gamma activity was suggested to be closely related to neuronal processing, especially in case of fMRI/EEG experiments high frequency EEG activity should be interpreted with caution since it is prone to be confounded by artifacts of non-neuronal origin (Muthukumaraswamy, 2013).

Similarly to the idea that the DMN is related to the combined activity of several distinct EEG microstates (Pascual-Marqui et al., 2014) and EEG frequency bands (Mantini et al., 2007; Chen et al., 2008; Neuner et al., 2014), it appears that several frequency bands within different EEG ICs contribute to the activity of the DMN.

The alpha rhythm has been the most widely studied brain rhythm in simultaneous EEG/fMRI research (Goldman et al., 2002; Laufs et al., 2003a; Gonçalves et al., 2006; de Munck et al., 2008). However, regarding the relationship between alpha power and the DMN, the results are largely inconclusive. In case of our study, we did not find an association between alpha power from any of the IC clusters with the DMN. Since the EEG spectral power and scalp distribution differs between eyes open and eyes closed states (Berger, 1934), it is possible that the alpha power to DMN relationship differs between these states. Mo et al. (2013) used occipital alpha activity to compare eyes open and eyes closed rest in a similar analysis of concurrent fMRI- and EEG-data. They only found an association with the DMN in eyes open rest, which confirms our findings, since in our eyes-closed paradigm no correlation with the alpha bands above the selected threshold was found.

### Replication of Previous Findings in Frontal (Midline) Theta

The term frontal midline theta can be traced back to the seventies (Ishihara and Yoshii, 1972) and refers to theta oscillations recorded at medial-frontal electrode positions. Subsequent investigations have linked frontal theta to working memory, episodic memory tasks and sustained cognition in general (Scheeringa et al., 2008; Michels et al., 2010; White et al., 2013). Moreover, it is a possible mechanism for cognitive control (Cavanagh and Frank, 2014), which is supported by findings of enhanced activity in adept meditators during concentrative meditation and thus heightened cognitive control (Brandmeyer and Delorme, 2016). Our observation of an association between theta activity in the frontal Cluster (Cluster 10 at time-point 1 and Cluster 17 at time-point 2) and DMN deactivation are consistent with previous findings (Scheeringa et al., 2008; Marawar et al., 2017) and can thus be viewed as a replication.

### Eye Movements as Behavioral Marker of DMN Activity?

Surprisingly, we found a negative association between the DMN and theta activity within a frontal EEG component that is commonly interpreted as eye movement component (e.g., Rissling et al., 2014). Two possibilities are conceivable which explain this association. First, the relationship between the frontal component and the DMN may be dominantly behavioral. Slow eye movements may elicit EEG signals in the theta frequency range while the level of movement decreases when activity of the DMN increases, for example during episodes of mind wandering. Previous studies have shown that mind wandering is associated with the complexity of eye movements during reading (Rayner and Fischer, 1996; Uzzaman and Joordens, 2011) and to pupil diameter, gaze position, and blink rate in a focused attention task (Grandchamp et al., 2014). Furthermore, activation of the DMN is consistently observed during episodes of mind wandering (Christoff et al., 2009; Ellamil et al., 2016; Scheibner et al., 2017). Hence, it is possible that a decrease in eye movements is a marker for activity of the DMN. Different resting state conditions, such as eyes-fixed resting state, might produce different eye movements and have been shown to differ in test-retest reliability (Patriat et al., 2013). This might explain why we are the first to report this finding. Second, it may be that the frontal eye-blink component is confounded by theta activity originating from the cortex, which was shown to negatively correlate with the DMN (Scheeringa et al., 2008). Consequently, the causal relationship between spontaneous decreases in theta activity from a frontal “eye movement” component and DMN activity needs further investigation.

### Limitations of This Study

We aimed for a primarily data-driven EEG IC clustering with minimal intervention to ensure reproducibility between the two time-points. One issue may be the inter-individual variability in components obtained from each subject and the potential assignment of several components per subject to each cluster. The decomposition into maximally ICs is inherently unstable and might be further destabilized by residual artifacts in the EEG data. This problem may be dealt with by application of methods that characterize the reliability of ICA decompositions within subjects (i.e., RELICA; Artoni et al., 2014). Furthermore, the number of clusters was chosen based on the amount of available components in each subject. This choice might be further improved by application of statistical indicators for the ideal number of clusters (Caliński and Harabasz, 1974).

A standard spherical 4-shell head model for the localization of dipoles associated with each IC was used and the spatial location of each dipole was included as criterion for the identification of similar ICs across subjects. However, it was shown that realistic head models which incorporate information about the head morphology, volume conductivity of the different tissue classes and exact electrode locations are important for an accurate EEG source localization (Ramon et al., 2006; Akalin Acar and Makeig, 2013; Cho et al., 2015). When accurate source localization is performed it is possible to detect RSNs from EEG data alone, which are comparable to the ones known from fMRI studies (Liu et al., 2017). Moreover, only components were allowed, whose equivalent dipole could explain more than 85% of the spatial variance and was located in the brain. While this criterion is widely used to ensure that components reflect brain sources, it could be invalid when looking for indicators of network activity, whose constituents are distributed across the brain.

Even though not formally assessed in this study, several average components appear similar at the two time-points, and the overall amount of components included in each cluster remained stable, which indicate some stability in the EEG ICA decompositions across time. Nevertheless, not all clusters could be identified at both time-points, which indicates that further investigation into the area of stable ICA decomposition and group-level component selection is needed. Approaches that aim to reduce the inter-individual variability in the group EEG data decomposition currently do not allow the application on resting state data (Eichele et al., 2011; Bigdely-Shamlo et al., 2013), but with advancing methodologies the comparability of ICs across subjects may greatly improve.

Besides differences induced by ICA decomposition and component clustering, behavioral changes may be associated with the difference in results. First, we did not control for the level of wakefulness during the resting state recording and it is possible that the subjects fell asleep during this time, influencing the association between EEG power and fMRI DMN. Second, the data we used were acquired as part of a meditation study, where two thirds of the participants underwent meditation training. Hence, it may be possible that the relation between EEG and fMRI activity has changed. We tested the subgroups with different meditation experience for differences in the topology of the DMN and found no significant deviations depending on amounts of meditation practice. However, it cannot be ruled out that differences in DMN activity and connectivity with other networks could have been present and even influenced the correlation between DMN BOLD fluctuations and EEG component activity. In our study, we did not address a possible difference but focused on general associations irrespective of the meditation experience that varied in our convenience sample. A larger sample size for subgroups would be required to investigate possible differences related to meditation practice.

In our analyses we created one GLM for each frequency band and IC, because the different frequency band power fluctuations can be significantly correlated with each other and we aimed to identify EEG parameters that could be used in EEG neurofeedback. Hence, we cannot distinguish the BOLD effect specific to each rhythm. Therefore, it would be interesting to investigate the specificity of EEG rhythms of different ICs and their association to the BOLD signal, while controlling for covariation between frequencies (Tyvaert et al., 2008; de Munck et al., 2009). Having said that, the correlation maps of Cluster 1, which corresponded to theta and beta1 power fluctuations, had opposite signs at time-point 1 and were reproduced at time-point 2. This shows that even if there is considerable cross-frequency correlation between neighboring frequency bands, differentiation between frequency bands further apart was achieved.

Although our results at time-point 1 are highly significant, the effect sizes are small to medium and the explained variance ranges from 5% to 12%. In a recent study, Yuan et al. (2016) attempted to reconstruct fMRI RSNs based on ICA decomposition. Instead of decomposing channel data, they temporally concatenated estimated amplitudes of current densities in distributed dipole sources and obtained spatial correlations in agreement with our results (Table 2, *r* = 0.28 correlation between the best matched EEG resting state component and the fMRI derived DMN). In our fMRI ICA the number of components was set to produce one DMN component. Allowing the DMN to split into several subcomponents might fit better to the dipole model that underlies the EEG ICA equivalent dipole model for quality control. Hence, the low spatial correspondence between networks independently derived from fMRI and EEG data indicates methodological difficulties when investigating the BOLD correlates of electrophysiological sources.

At the end, our results show stable relationships between several IC clusters and the DMN in our sample. This is an important step in the search for reliable EEG parameters that indicate RSN activity and thus for targeting specific networks with EEG-neurofeedback.

## Conclusion

In conclusion, our results indicate that the DMN as seen in fMRI is reflected in a combination of different frequency bands within several EEG ICs. One major strength of our study is the replication of the analysis with data collected from the same subjects 1 year apart, which indicated a stable relationship between several EEG ICs and fMRI-BOLD activity during rest. Furthermore, the results from an EEG “eye movement” component may provide useful as a behavioral marker of DMN activity. In addition to previous studies investigating the BOLD correlates of scalp level EEG activity, we attempted to increase the specificity by decomposition of the EEG signal into temporally ICs. We were able to recover the DMN independently using two different approaches, a group ICA on concatenated multi-subject fMRI data and based on frequency separated EEG data in a regression model.

## Author Contributions

MT and UO collected the data. UO developed the idea for data analysis. UO, TS and MP analyzed the data. TS and MP wrote the manuscript. MT, RS and UO gave feedback on manuscript. TS and MP contributed equally.

## Conflict of Interest Statement

The authors declare that the research was conducted in the absence of any commercial or financial relationships that could be construed as a potential conflict of interest.

## Footnotes

^1^http://www.sccn.ucsd.edu/eeglab

^2^http://www.fil.ion.ucl.ac.uk/spm/software/spm12

^3^www.fmrib.ox.ac.uk/fsl

^4^http://www.fmrib.ox.ac.uk/datasets/brainmap+rsns
